# Proactive Identification of Patients with Diabetes at Risk of Uncontrolled Outcomes during a Diabetes Management Program: Conceptualization and Development Study Using Machine Learning

**DOI:** 10.2196/54373

**Published:** 2024-04-26

**Authors:** Arash Khalilnejad, Ruo-Ting Sun, Tejaswi Kompala, Stefanie Painter, Roberta James, Yajuan Wang

**Affiliations:** 1 Teladoc Health Purchase, NY United States

**Keywords:** diabetes, diabetic, DM, diabetes mellitus, type 2 diabetes, type 1 diabetes, self-monitoring, predictive model, predictive models, predictive analytics, predictive system, practical model, practical models, ML, machine learning, AI, artificial intelligence, algorithm, algorithms, behavior, behaviour, telehealth, tele-health, chronic condition, chronic conditions, chronic disease, chronic diseases, chronic illness, chronic illnesses

## Abstract

**Background:**

The growth in the capabilities of telehealth have made it possible to identify individuals with a higher risk of uncontrolled diabetes and provide them with targeted support and resources to help them manage their condition. Thus, predictive modeling has emerged as a valuable tool for the advancement of diabetes management.

**Objective:**

This study aimed to conceptualize and develop a novel machine learning (ML) approach to proactively identify participants enrolled in a remote diabetes monitoring program (RDMP) who were at risk of uncontrolled diabetes at 12 months in the program.

**Methods:**

Registry data from the Livongo for Diabetes RDMP were used to design separate dynamic predictive ML models to predict participant outcomes at each monthly checkpoint of the participants’ program journey (month-n models) from the first day of onboarding (month-0 model) up to the 11th month (month-11 model). A participant’s program journey began upon onboarding into the RDMP and monitoring their own blood glucose (BG) levels through the RDMP-provided BG meter. Each participant passed through 12 predicative models through their first year enrolled in the RDMP. Four categories of participant attributes (ie, survey data, BG data, medication fills, and health signals) were used for feature construction. The models were trained using the light gradient boosting machine and underwent hyperparameter tuning. The performance of the models was evaluated using standard metrics, including precision, recall, specificity, the area under the curve, the *F*_1_-score, and accuracy.

**Results:**

The ML models exhibited strong performance, accurately identifying observable at-risk participants, with recall ranging from 70% to 94% and precision from 40% to 88% across the 12-month program journey. Unobservable at-risk participants also showed promising performance, with recall ranging from 61% to 82% and precision from 42% to 61%. Overall, model performance improved as participants progressed through their program journey, demonstrating the importance of engagement data in predicting long-term clinical outcomes.

**Conclusions:**

This study explored the Livongo for Diabetes RDMP participants’ temporal and static attributes, identification of diabetes management patterns and characteristics, and their relationship to predict diabetes management outcomes. Proactive targeting ML models accurately identified participants at risk of uncontrolled diabetes with a high level of precision that was generalizable through future years within the RDMP. The ability to identify participants who are at risk at various time points throughout the program journey allows for personalized interventions to improve outcomes. This approach offers significant advancements in the feasibility of large-scale implementation in remote monitoring programs and can help prevent uncontrolled glycemic levels and diabetes-related complications. Future research should include the impact of significant changes that can affect a participant’s diabetes management.

## Introduction

Diabetes is a chronic disease that affects 37.3 million individuals living in the United States and requires ongoing management [1]. Common diabetes-related health complications that can be delayed or prevented with glycemic control include heart disease, chronic kidney disease, and neuropathy. Diabetes management is multifaceted and should include some level of provider care, medication management, and self-management. Telehealth interventions have been shown, and are encouraged, to support condition management and the reduction in diabetes-related complications [2]. With the current capabilities and advancements of telehealth in diabetes management, it is critical to help identify individuals with a higher risk of uncontrolled diabetes and provide them with targeted support and resources to help them manage their condition [3,4].

Predictive modeling has emerged as a valuable tool in diabetes management, offering numerous benefits for individuals with diabetes. By engaging early in a remote diabetes monitoring program (RDMP), predictive modeling can capture the user’s interest and motivation, fostering active participation in their own health through suggesting personalized behavior change in a timely manner to improve outcomes. The integration of proactive targeting into routine diabetes care also holds immense potential to transform the lives of millions affected by this chronic condition through preventing or delaying the onset of diabetes-related complications and reducing the burden on the health care system by lowering the cost of care and improving the quality of care. The current literature describing applied or developed predictive models has used insurance claims or electronic medical records to predict varying outcomes related to uncontrolled diabetes; however, there is a lack of literature on the prediction of uncontrolled diabetes management through a large data set of self-monitored blood glucose (SMBG) values and contributing factors, which provides a unique pathway to real-time predictions [5-7].

Machine learning (ML) can analyze a large amount of data and identify meaningful patterns that correspond to the diabetes risk level of individuals and predict their diabetes outcome risk [8-11]. The objective of this study was to conceptualize and develop a novel ML approach to proactively identify participants enrolled in a large-scale RDMP who were at risk of uncontrolled diabetes at 12 months in the program. Therefore, a set of dynamic predictive ML models were designed and trained at specific checkpoints during the participants’ time in the program to proactively identify those at risk and capture participant attributes that could impact the participants’ at-risk status.

## Methods

### Ethical Considerations

Approval was granted by the Aspire Institutional Review Board (IRB; #520160099), and guidelines outlined in the Declaration of Helsinki were followed. All participants provided consent to participate during enrollment into the Livongo for Diabetes RDMP, and guidelines outlined in the Declaration of Helsinki were followed. All study data were stored in Health Insurance Portability and Accountability Act–compliant secure servers and were deidentified prior to analysis. Participants were not compensated for their participation in the study.

### Livongo for Diabetes

Teladoc Health’s Livongo for Diabetes is an RDMP focused on empowering participants with education and tools to self-manage their diabetes through mobile technology. The program offers participants a cellular-enabled, 2-way messaging device that measures blood glucose (BG) levels and delivers personalized insights into their glycemic management; free unlimited BG test strips; real-time support from diabetes response specialists 24 hours a day, 7 days a week, 365 days a year; and access to certified diabetes care and education specialists (CDCESs) for support and goal setting.

Teladoc participants’ BG meter use was captured remotely through the cellular-enabled device. Participants also had access to a web-based app and mobile phone app that tracked historical SMBG readings; provided reminders for SMBG checking, physical activity (PA), and food log tracking; and provided an asynchronous chat with coaches, the ability to schedule private coaching sessions with CDCESs, educational content for diabetes self-management, and the ability to send historical reports of SMBG readings to care providers, family members, and friends.

### Diabetes Management Journey

The diabetes management journey (ie, program journey) refers to the ongoing process of monitoring and managing diabetes through the Livongo for Diabetes RDMP. The program journey involves education and support to make lifestyle changes, monitor SMBG levels, take medications as prescribed, and work with health care providers to achieve optimal health. The goal is to support participants with diabetes achieve BG control for complication avoidance and lead healthy, active lives.

### Study Design

Registry data from the RDMP were used to design separate ML models to predict participant outcomes at each monthly checkpoint of the program journey (month-n models) from the first day of onboarding (month-0 model) up to the 11th month (month-11 model). The program journey of a participant began upon onboarding into the Livongo for Diabetes RDMP and capturing their first BG measurement through a Livongo BG meter. Overall, each participant passed through 12 predicative models through their first year enrolled in the program.

### Measures

#### Hemoglobin A1c and Observability

Hemoglobin A1c (HbA1c), or glycated hemoglobin, is a critical metric for diabetes management, which provides a long-term picture of an individual’s average BG levels over a 2-3-month period [12]. HbA1c cannot be directly measured with a BG meter, which only measures the current BG level in the blood. To calculate HbA1c from BG meter readings, an algorithm that considers the average BG levels and the frequency of measurements was used: A1c = [average glucose (mg/dL) – 46.7]/28.7 [13]. In this algorithm, the more frequent and consistent the BG readings, the more accurate the estimate of A1c (eA1c). A participant was considered “observable” if enough BG readings over 90 preceding days provided statistical confidence to estimate clinically meaningful A1c, otherwise the participant was considered “unobservable.” Statistical significance was determined by considering both the mean and SD of BG checks. The more variable the set of BG checks for a given participant, the greater the threshold for that participant to be deemed observable.

### Population Selection

Participants enrolled in the Livongo for Diabetes RDMP between January 1, 2019, and January 1, 2022, with an activated BG meter who met the criteria to be categorized as observable at month 12 in the program were included as study participants (N>200,000)*.* Ground truth labeling as “cases” and “controls” for diabetes management conditions was performed using 12-month eA1c values as follows: (1) participants with month 12 eA1c≥7.5% were labeled as cases and defined as participants at risk of uncontrolled diabetes management outcomes and (2) participants with month 12 eA1c<7.5% were labeled as controls and defined as participants not at risk of uncontrolled diabetes management outcomes. The ratio of cases to controls in the study population was 23.5%-76.5%.

### Model Design: Participant Features and Attributes

Using diabetes-related available features and attributes of participants through their program journey, each ML model was designed and developed to make a binary prediction of whether the participant will be at risk of uncontrolled diabetes at the end of month 12 in their program journey. The eA1c at month 12 was used to generate binary labels for either month 12 eA1c≥7.5% or month 12 eA1c<7.5% as controls.

During the program journey, participants used the BG meter provided to record BG levels. Next, A1c was estimated using the accumulation of BG levels over a 90-day period. As described previously, clinically meaningful calculation of a member’s eA1c is dependent on the classification of observability. Since A1c has remained the clinical gold standard for indexing chronic glycemia for decades, and eA1c is an essential metric in assessing a participant’s diabetes condition, observability was a critical metric to predict participant outcomes. For unobservable participants, due to the lack of eA1c related to sparsity of BG checks and with the availability of derived program features, other features and attributes played a significantly more important role in the predictive ML models. Therefore, at each month of predictive modeling design, it was essential to train 2 separate models to cover participants with observable and unobservable eA1c values, making a total of 24 trained models.

The goal of an ML model is to find and learn patterns of input features from training data and then use them to make predictions on new, unseen data. Therefore, the quality and relevance of the features used are crucial for the performance of an ML model. We trained a set of ML models that needed to be sequentially compared, which made it critical to keep the structure of features consistent along the program journey to obtain a robust interpretation of features evolving among models.

#### Participant Attribute Categories

Participant attributes, including survey data, BG data, medication fill data, and health signals, were used for feature construction within the ML models.

#### Survey Data

Survey data gathered from the participants at enrollment included demographic information, such as age, gender, race, ethnicity, height, weight, BMI, language, and diabetes type. Participant intention and preference information around the diabetes management style, interest in becoming more active, and interest in healthy eating was also included in survey data. Each of the engagement attributes was encoded for model features as ordinal values. For example, in the case of interest in healthy eating, the options of not important, somewhat important, and very important were encoded as 1, 2, and 3, respectively.

#### Blood Glucose Data

BG data were measured through RDMP-provided BG meters with blood from a finger prick applied to a test strip, and participants were asked to select “feel tags” and “meal tags” from a set of options. Features were constructed from an accumulation of SMBG readings by 30-day aggregates, BG readings broken down by meal and feel tags, and A1c. The 30-day BG check aggregates generated data of the total number of readings and the number of hypoglycemic and hyperglycemic BG levels. Since the ML models were designed in monthly checkpoints, 30-day aggregates were important indicators of a participant’s diabetes pattern change.

BG levels are affected by both diet and mental health; therefore, BG readings were broken down by meal and feel tags to be correlated along each BG reading [14-16]. Meals increase glucose levels, while fasting typically decreases BG levels. BG levels can also be impacted by feelings, such as stress, and PA. Therefore, in the features, BG levels were broken down based on meal and feel tags. The meal tag options include before/after breakfast, before/after lunch, before/after dinner, after snack, and no meal. In addition, the feel tag options include feel fine, feel sick, stressed, ate extra, lightheaded, after exercise, missed medications, increased medications, and other feelings.

A1c is an important feature in diabetes management as it provides a snapshot of an individual’s overall BG control. In addition, variability of A1c depicts the diabetes management condition of an individual over time [17]. In the RDMP, A1c was estimated if a participant was observable. This feature was only available for observable participants, who recorded enough SMBG readings to estimate A1c with statistical significance.

#### Medication Fill Data

A GPI is a unique identifier assigned to a drug product to distinguish it from similar products and improve identification and tracking in the health care supply chain. Generic product identifiers (GPIs) were tracked over time to capture medicine fills, understand medication adherence, and determine a change in the health status of participants related to their diabetes. Based on participants’ medicine fills, they were categorized into the following diabetes groups using medication use as a proxy for disease progression [18]:

- Group 1: participants with type 2 diabetes who use only lifestyle modification (diet and exercise) and take no medication
- Group 2: participants with type 2 diabetes who take metformin
- Group 3: participants with type 2 diabetes who take metformin and other noninsulin medications
- Group 4: participants with type 2 diabetes who use basal insulin, in addition to other medications
- Group 5: participants with type 2 diabetes who use basal and bolus insulin (also known as multiple daily injections [MDIs] or intensive insulin)
- Group 6: participants with type 1 diabetes

#### Health Signal Data

A health signal refers to any measurable aspect of an individual’s physical or physiological state that provides information about their health status. Based on availability, 30-day aggregates of health signals were used in the features, such as average systolic and diastolic blood pressure (BP) readings, whether participants were enrolled in the Livongo for Hypertension (HTN) program, and the average weight, average daily PA, and food logs if participants were enrolled in weight management (WM).

Tracking these features that contribute to participant attribute categories over time can assist in personalized effective interventions for at-risk observable and unobservable participants.

### Outcome Data

During evaluation of performance of the ML models for identification of participants at risk of uncontrolled diabetes management outcomes, various viewpoints were considered to prevent model shortcomings and imbalanced data: (1) How many at-risk participants were identified and with what precision by each model? (2) How many participants were targeted to achieve the performance metrics? (3) How does the progression of the performance metrics look over a participant’s program journey?

To provide a comprehensive view of model performance and address the aforementioned questions, the following 6 measures commonly used in ML model evaluation were selected [19]:

- Sensitivity or recall: Recall is a measure of how well a model can identify positive (at-risk participants) instances defined as the number of true-positive predictions divided by the total number of positive instances in the data set.
- Precision: Precision is a measure that evaluates the proportion of positive predictions that are correct and defined as the number of true-positive predictions divided by the sum of true-positive predictions and false-positive predictions.
- Specificity: Specificity is a measure that evaluates the ability of a model to correctly identify negative (not-at-risk participants) examples. It is the proportion of true negatives over all negatives.
- Area under the curve (AUC): The AUC represents a model’s ability to distinguish between positive and negative examples. In this study, AUC values ranged from 0 to 1, with a value of 0.5 representing a random guess and a value of 1 representing a perfect model. An AUC of 0.7 or higher is generally considered good, while an AUC of 0.9 or higer is considered excellent.
- *F*_1_-score: The *F*_1_-score is a performance metric that balances the precision and recall by calculating the harmonic mean of these 2 metrics.
- Accuracy: Accuracy measures the proportion of correct predictions made by a model out of all predictions.

### Model Development

For proactive targeting of the RDMP participants’ diabetes management outcomes, it was necessary to train a set of ML models at specific time stamps through each participant’s program journey for the following reasons. First, the diabetes condition, such as severity, complications, and medication use, of a participant can evolve over time and impact the outcome. Similarly, the diabetes management patterns of participants, such as exercise, diet, and SMBG checking patterns, can alter over time and change the outcome trajectory. To capture these changes, new models needed to be trained along the program journey to update the risk prediction using more recent accumulated information. Second, as participants progressed in their program journey, more temporal data were collected from their retrospective diabetes management attributes. This change in the quality of attributes can change their importance and contribution in the ML modeling, indicating the need for new model training along the participant program journey.

**Figure 1:**
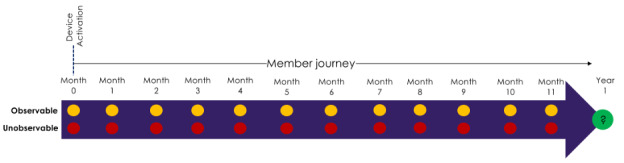
Diabetes proactive targeting framework at each monthly observable and unobservable segment of each participant’s program journey. The program journey starts from device activation, and at each monthly checkpoint, based on the participant’s observability status, an ML model is trained to predict the outcome of the diabetes condition of the participant at year 1 of the program journey. ML: machine learning.

As previously mentioned, from the first day of Livongo BG device activation, at each monthly step, a prediction ML model was trained to predict the binary outcome of the diabetes management status of each participant at 12 months in the program. At each month of the program journey, there were 2 separate models to develop based on observability. Figure 1 represents the timeline of modeling work for observable and unobservable segments of the participants’ program journey. Based on the designed framework, a set of 24 ML models were trained to capture the relationship between participants’ input attributes and diabetes management control outcomes.

In modeling, for each model, data were split into 2 subsets. The first subset, which contained a larger portion of the data, was used as training and validation data to train the model and tune the hyperparameters, as well as find the optimal parameters of the model to achieve the highest accuracy. The second subset was used as testing data, which was held separate from the training data and used to assess how well the model could work with new data. Each model randomly split the data into 85% training and 15% testing subsets.

The models were based on light gradient boosting machine (LightGBM), which is a tree-based learning algorithm developed on the randomly selected subset of training data. With 5-fold cross-validation on training data, the Hyperopt Python library was used for tuning the hyperparameters of each LightGBM model, including the number of estimators, learning rate, number of leaves, feature fraction, and bagging fraction [20,21]. The tree-structured Parzen estimator (TPE) algorithm was used to optimize hyperparameter quantization [22]. Finally, the performance of each model was assessed on the unseen test data sets.

Training and testing instances for cases and controls among the data subsets for observable and unobservable participants for each month of the program journey were distributed (see Multimedia Appendix 1). The class imbalance of observable and unobservable participants was on average 78% versus 22% and 69% versus 31%, respectively, for controls and cases. To mitigate this highly imbalanced ratio of controls and cases and increase the focus of the models on less prevalent cases, the models were set to assign class weights inversely proportional to their respective frequencies during the training process.

### Bias Mitigation Consideration

Bias reduction in ML models is crucial to ensure fair and equitable outcomes. The following considerations were taken to reduce bias in the ML model: class imbalance handling, feature selection and inclusion, observability consideration, missing imputation, cross-validation, and temporal changes.

#### Class Imbalance Handling

As previously mentioned, there was a class imbalance in the observable and unobservable participants. To address this, the models were set to assign class weights inversely proportional to their respective frequencies during the training process. This technique helped the models give more importance to the minority class, reducing bias toward the majority class.

#### Feature Selection and Inclusion

Four categories of participant attributes for feature construction within the models were used: survey data, BG data, medication fills, and health signals. This diverse set of features helped in capturing different aspects of participants’ health and behavior, reducing bias that might have arisen from relying on a limited set of features.

#### Observability Consideration

The observability of participants, distinguishing between observable and unobservable participants, was used. This factor was explicitly considered during model development, with different models trained for observable and unobservable participants. This approach acknowledged and addressed the potential bias introduced by the availability of certain data for only a subset of participants.

#### Missing Imputation

For unobservable participants with missing eA1c values, a robust imputation approach was used, which involved a mixture of historical eA1c data interpolation, leveraging their past records, and incorporation of similarity features from other participants, considering factors such as age and diabetes medications. This approach aimed at reducing bias in the imputation process, ensuring a more accurate estimation of missing eA1c values.

#### Cross-Validation

The models were based on LightGBM and underwent hyperparameter tuning using 5-fold cross-validation on training data. Cross-validation helped in assessing each model’s performance across different subsets of the data, reducing the risk of overfitting and ensuring generalizability.

#### Consideration of Temporal Changes

The study acknowledged the dynamic nature of diabetes conditions and the importance of capturing temporal changes. The models were trained at specific checkpoints during each participant’s program journey, allowing the models to adapt to evolving patterns and reducing bias introduced by changes over time.

### Summary of the Methodology Workflow

**Figure 2:**
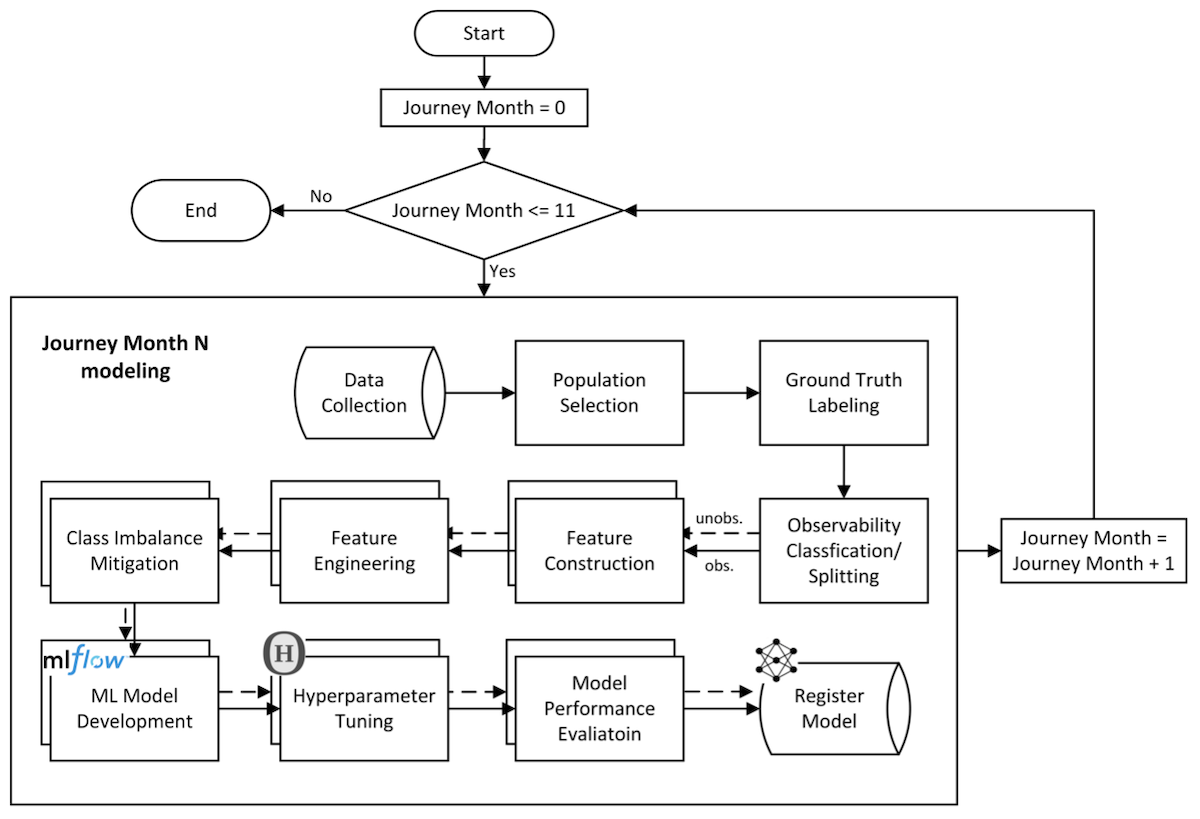
Sequential steps in the modeling workflow, which included data preprocessing, feature preparation, model training, and model registry at each monthly program journey checkpoint. ML: machine learning; obs.: observed; unobs.: unobserved.

The detailed sequential steps of the modeling process are listed next and represented visually in Figure 2:

- Step 1 (data collection): Registry data from the Livongo for Diabetes RDMP were collected.
- Step 2 (population selection): Participants with an activated BG meter who met criteria for observability at month 12 were selected.
- Step 3 (ground truth labeling): Participants with eA1c≥7.5% were labeled as cases, and those with eA1c<7.5% were labeled as controls.
- Step 4 (observability classification/splitting): Participants were classified as observable or unobservable based on the statistical confidence derived from a sufficient number of BG readings over the preceding 90 days.
- Step 5 (feature construction): Features were constructed based on survey data, BG reading breakdown by meal and feel tags, medicine fill data, and health signals.
- Step 6 (feature engineering): Features were transformed to accommodate high performance of ML modeling. These steps included missing imputation using a combination of historical data and relevant participant characteristics with normalization and standardization of numerical features. In addition, for categorical features, ordinal encoding was applied, when possible. When ordinal encoding was not possible, one-hot-encoding was performed.
- Step 7 (class imbalance mitigation): To address imbalance between controls and cases, class weights were assigned inversely proportional to their respective frequencies during the training process.
- Step 8 (ML model development): Separate ML models were designed and trained at specific checkpoints during each participant’s program journey (from month 0 to month 11) for both observable and unobservable segments.
- Step 9 (hyperparameter tuning): Hyperparameter tuning was performed using the Hyperopt Python library, optimizing parameters such as the number of estimators, learning rate, number of leaves, feature fraction, and bagging fraction.
- Step 8 (model performance evaluation): Models were evaluated using standard metrics, including precision, recall, specificity, the AUC, the *F*_1_-score, and accuracy.
- Step 10 (register models): Each trained model was registered into the model registry to be used for inference.

### Descriptive Analysis

Patterns and trends of static and time series features and their correlation to the outcomes represented the participant breakdown by interest in learning about healthy eating and interest in becoming more active from the participant onboarding survey along with 12-month eA1c values. Assessing mean BG levels by meal and feel tags was crucial for predictive modeling to provide valuable insights into the patterns and factors influencing diabetes management outcomes. Understanding how BG levels vary before and after meals and in relation to different feelings supports the identification of critical points in a participant’s daily routine that may contribute to uncontrolled outcomes.

### Statistical Analysis

To examine the correlation strength between extracted features and the diabetes outcomes (uncontrolled/controlled), Pearson r correlation analysis was used and represented top performers among 4 categories of attributes.

## Results

### Participant Demographics

Participant demographics and characteristics at the time of program enrollment are presented in Table 1.

**Table 1:** Participant demographics and characteristics at the time of enrollment into the Livongo for Diabetes RDMP^a^.

Characteristics		Participants
**Diabetes type, %**		
	Type 2 diabetes	92.1
	Type 1 diabetes	7.3
	Unknown	0.5
**Insulin use, %**		
	No	78.8
	Yes, once/day	13.1
	Yes, more than once/day	8.0
	Unknown	0
**Race, %**		
	Unknown	47.9
	White/Caucasian	37.1
	Black/African American	7.4
	Asian/Chinese/Japanese/Korean	3.9
	Other	3.1
	American Indian or Alaskan Native	0.4
	Native Hawaiian or Other Pacific Islander	0.2
**Ethnicity, %**		
	Unknown	47.7
	Non-Hispanic	46.3
	Hispanic	6.0
**Gender, %**		
	Male	51.7
	Female	48.3
Age (years), mean (SD)		61.6 (12.3)
Year since diabetes diagnosis, mean (SD)		9.03 (9.19)
Self-reported A1c at enrollment, mean (SD)		7.33 (1.54)

^a^RDMP: diabetes remote monitoring program.

### Descriptive and Statistical Analysis

**Figure 3:**
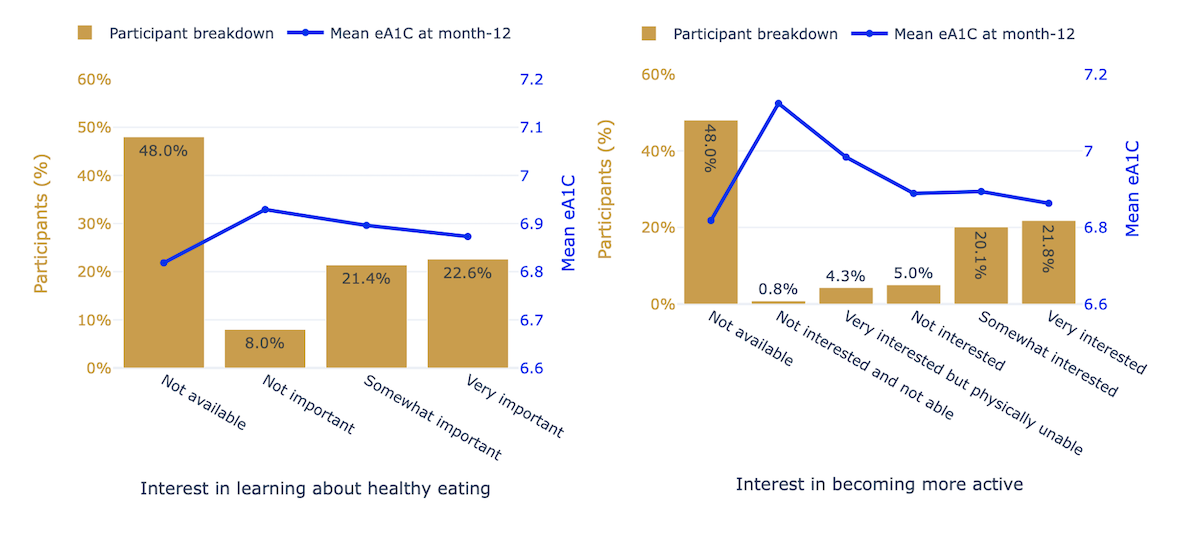
Participant breakdown and mean eA1c of survey responses to (left) interest in learning about healthy eating and (right) interest in becoming more active. eA1c: estimate of A1c.

Patterns and trends of static and time series features and their correlation to the outcomes are illustrated in Figure 3. Figure 3a shows that participants with a higher interest in learning about healthy eating had a lower mean eA1c outcome. Similarly, as shown in Figure 3b, participants with a higher interest in becoming more active had a lower mean eA1c outcome.

**Figure 4:**
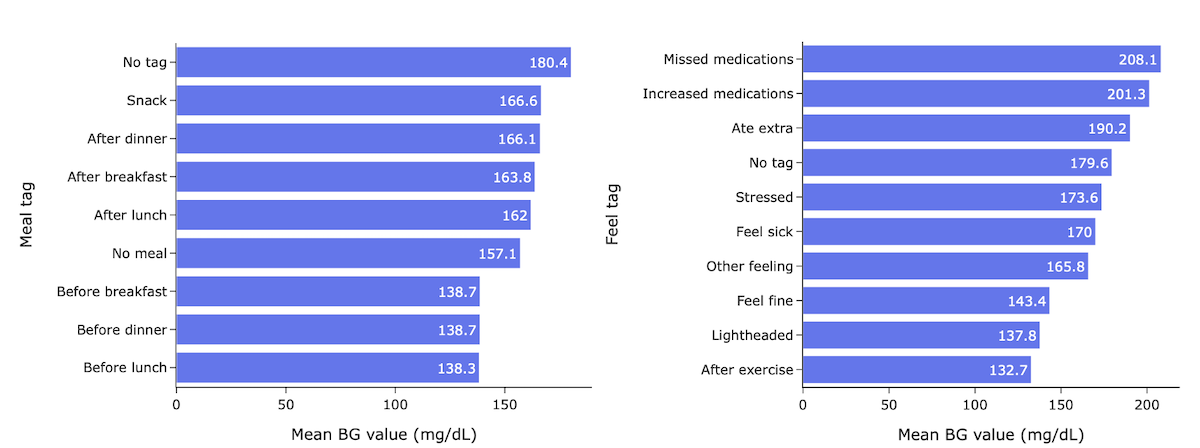
Mean BG level of participants along their 1-year program journey by (left) meal tag and (right) feel tag. BG: blood glucose.

Figure 4 represents the mean BG levels of participants along their 1-year program journey by meal tag and feel tag. As shown in Figure 4a, the BG level tagged after breakfast, lunch, or dinner was on average 20% higher than that before meals. In addition, as illustrated in Figure 4b, BG levels related to feeling fine, lightheaded, and after exercise were the lowest, while those after missed or increased medications and eating extra were the highest.

In the BG data category, the BG check breakdown by feel and meal tags was highly correlated, including BG checks tagged as “feel fine” (*r*=–0.18, *P*<.001) and before breakfast, lunch, and dinner (*r*=0.155, *P*<.001). In addition, among survey data, age had the highest correlation (*r*=0.164, *P*<.001). Among medicine fills, the metformin fills feature was presented as a top feature (*r*=0.072, *P*<.001). Finally, among health signals, 30-day PA had a correlation of 0.039 (*P*<.001).

### Performance Results and Output of the 12 ML Models

Performance results of the 12 ML models for observable participants for the training and testing subsets from month 0 to month 11 of their program journey are presented in Multimedia Appendix 2. It was crucial to understand that due to class imbalance, a random prediction would not result in a theoretical precision or an *F*_1_-score of 0.5. The precision obtained from a random prediction would be proportional to the number of cases, and the *F*_1_-score would be accordingly impacted. For observable participants, the baseline case was on average 0.22 (range –0.009 to +0.009 across months in the program journey), which was outperformed by the precision of monthly models with an average precision of 61%, ranging from 0.4 at month 0 to 0.88 at month 11, with consistent improvement along the participant’s program journey. Similarly, recall started from 0.7 at month 0 of the program journey and reached 0.94 at month 11. The AUC of the models also improved consistently, ranging from 0.76 to 0.98 across models along the participant’s program journey.

Similar to observable participants, results of the performance of the 12 ML models for unobservable participants along their program journey are shown in Multimedia Appendix 3. For unobservable participants, the baseline case was on average 0.31 (range –0.05 to +0.035 across months in the program journey), which was outperformed by the precision of monthly models with an average precision of 0.55, ranging from 0.42 at month 0 to 0.61 at month 11 on the testing set.

The recall of the ML models at month 0 was 0.61 and by further progress of the participant’s program journey increased to 0.82, with a precision of 0.61 at month 11. At month 1, there was a significant improvement in performance, with an improved recall and precision increase of +0.17 and +0.11, respectively. This difference was due to the difference in input features of month 0 and other monthly models. At month 0, only survey data and medicine fills were being used as model features; however, for the rest of the models, BG data and health signals were added, causing a drastic improvement in the performance of the models. In addition, the AUC of the models was on average 0.8 (range 0.7-0.87), representing good performance of the models.

**Figure 5:**
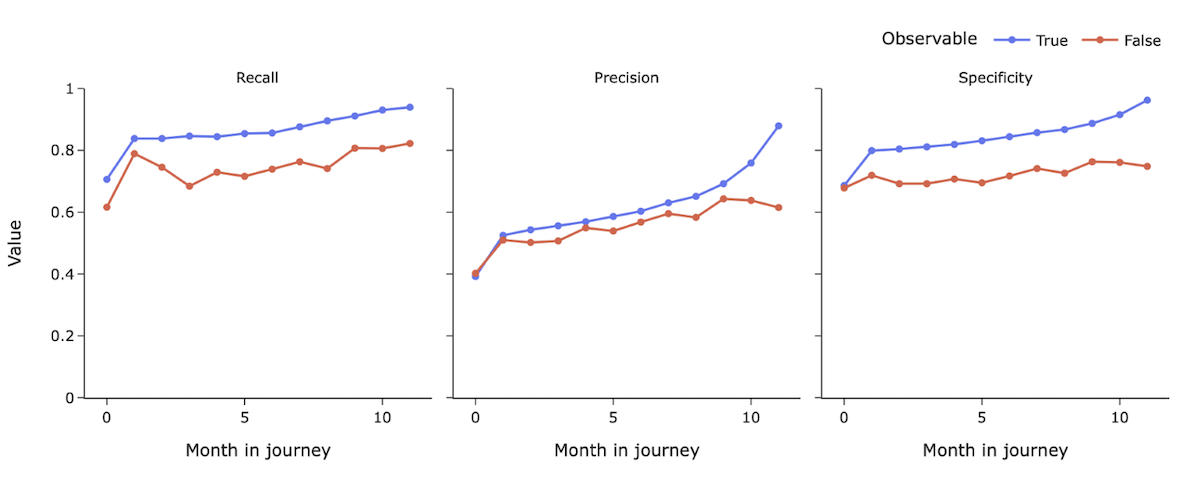
Performance metrics (recall, precision, and specificity) of models along each participant’s program journey for observable (blue) and unobservable (red) subsets.

Figure 5 represents the comparative performance of the models along the monthly program journey for observable and unobservable participants on the testing sets. In the figure, blue and red lines represent the performance metrics for observable and unobservable participants, respectively. On average, the recall of models for observable participants was 0.09 higher than that of models for unobservable participants, with the difference ranging from 0.04 to 0.16. Similarly, the precision of models for observable participants was on average 0.06 higher than that of models for unobservable participants, with a range of 0.02-0.26. In addition, specificity metrics had the highest difference in these 2 groups, with an average difference of 0.11.

## Discussion

### Principal Findings

The ML approach used in this study demonstrated high capability to proactively identify participants enrolled in an RDMP who were at risk of uncontrolled diabetes using participant survey inputs, SMBG data, medical history, and diabetes management engagement signals. Proactive targeting models accurately identified observable participants at risk of uncontrolled diabetes (71%-94%; mean 86%, SD 6%) and unobservable at-risk participants (64%-82%; mean 77%, SD 5%) from month 0 to month 11, with an achieved precision of 40%-88% (mean 62%, SD 12%) and 42%-61% (mean 57%, SD 8%), respectively. As participants progressed through their program journey, the prediction models became more accurate and performed better in identification of those at risk among observable participants compared to unobservable participants. In addition, the performance difference between month 0 and month 1 in both observable and unobservable participants was significantly higher than in the consecutive months, demonstrating the importance of engagement data for predicting long-term clinical outcomes. The most critical feature in the models was the last-available eA1c, followed by diabetes-related medication fills and BG checking patterns with meal and feel tags [23].

### Comparison With Prior Work

Various prediction models have been developed and implemented in the literature over the past decade with increased accuracy in predicting the diabetes risk over time, specifically the transition of prediabetes to diabetes [11,24,25]. However, nearly all the literature focuses on health care data sets from hospital patients [10,26,27]. Although these studies have shown that ML models can preserve performance across populations with health care data collected through demographic data, laboratory values, and hospital records, there is a lack of literature implementing ML and proactive targeting in a real-world RDMP predicting an individual with diabetes entering uncontrolled diabetes status using engagement behaviors and SMBG levels. Additionally, prior studies restrict input data in their models to contain specific information, such as baseline A1c, and train models on a specific cohort of uncontrolled baseline A1c [28]. Our study focused on ML models with the ability to perform on limited and sparse data that are indicative of diabetes management in a real-world population.

The data available through the Livongo for Diabetes RDMP supported the ability to develop and achieve significant accuracy of the ML models used in this study. Consistent capture of participants’ SMBG patterns and frequency and self-monitoring behaviors around PA, diet, and mental health was essential to develop models for both observable and unobservable participants. By conducting sufficient SMBG checks with statistical confidence, a participant’s A1c can be estimated and the participant becomes observable, which was an important parameter in our study. Due to fewer data points of SMBG-related features for unobservable participants, other features became more important in their corresponding predictive models. The result of this study’s approach is applicable to proceeding years of participants’ program journey and other chronic condition remote monitoring program journeys.

The results achieved in this study demonstrate robust and generalizable performance in proactive targeting of at-risk participants enrolled in an RDMP, which can provide significant advancements toward the feasibility of large-scale implementation in the following aspects. First, early identification of at-risk participants will allow for ease in effective interventions to change the trajectory of outcomes and aid in the prevention of potential uncontrolled glycemic levels and diabetes-related complications. Second, provided that participants can become observable or unobservable and at risk or not at risk at varying time points throughout the program journey, proactive targeting will ensure identification of participants with higher probability. Lastly, using proactive targeting, along predictive models, the evolving metrics for diabetes management can also be obtained, such as the diabetes medication stage. These metrics combined with predictive outcomes can provide personalized intervention strategies to improve participant outcomes.

### Strengths and Limitations

This study has many strengths, including the use of data collected from participants enrolled in a real-world RDMP. The ML models used in the study also have 2 distinguishing strengths. First, the proactive targeting design enables ongoing tracking of participants’ diabetes management condition toward reaching the desired outcome and early identification of at-risk participants, which allows for identification of factors that may cause a participant to become at risk of uncontrolled diabetes. Second, the models are not limited to the availability of specific data. In fact, the lack of availability and sparsity in each feature can represent an important factor about the participants. For example, the sparsity of BG checks can represent low program engagement, especially for participants with higher diabetes severity. This consideration avoids the self-selection bias of the models toward active participants.

Some limitations also exist related to the complexity of identifying participants at risk of uncontrolled diabetes management outcomes proactively. Due to the signal of SMBG levels being dependent on SMBG checking patterns of participants, the accuracy of prediction decreases by participant inactivity. This limitation can be resolved by using other BG monitoring technologies, such as continuous glucose monitoring devices [29]. In addition, identifying at-risk participants earlier within the program journey would be helpful to develop effective and timely interventions; however, the lower performance of models at earlier time points due to a lack of enriched features limited this objective. In addition, the models may have been influenced by external factors not accounted for in our study, such as individual socioeconomic factors and environmental variables. This issue can be partially diminished by adding extra generalizable features, such as the social determinants of health, to the feature sets [30]. Future research could explore the incorporation of a broader range of contextual features to enhance the models’ robustness and generalizability across diverse populations.

### Conclusion

This study explored participants’ temporal and static attributes, the identification of diabetes management patterns and characteristics, and their relationship to predict diabetes management outcomes. Proactive targeting models accurately identified participants at risk of uncontrolled diabetes with a high level of precision that was generalizable through future years within the RDMP. Future research should include the impact of significant changes that can affect a participant’s diabetes management—for example, granular medication changes, such as drug and dosage changes, as well as medication adherence, which are important factors for the determination of diabetes outcomes [31].

## Appendix

Multimedia Appendix 1 Number of participant cases and controls for training and testing the data subset for each monthly checkpoint of the participants’ program journey.

Multimedia Appendix 2 Performance metrics for training and testing the data subset for observable participants at each monthly checkpoint of their program journey. The accuracy metrics for each month in the program journey represent the performance of a stand-alone ML model. ML: machine learning.

Multimedia Appendix 3 Performance metrics for training and testing the data subset for unobservable participants at each monthly checkpoint of their program journey. The accuracy metrics for each month in the program journey represent the performance of a stand-alone ML model. ML: machine learning.

## Abbreviations

AUC: area under the curve
BG: blood glucose
CDCES: certified diabetes care and education specialists
eA1c: estimate of A1c
GPI: generic product identifier
LightGBM: light gradient boosting machine
ML: machine learning
PA: physical activity
RDMP: remote diabetes monitoring program
SMBG: self-monitoring blood glucose

## Data Availability

Deidentified data from this study are not available in a public archive and will be made available (as allowable according to Institutional Review Board standards) by emailing the corresponding author.
