# Supplementary material for: Proactive Identification of Patients with Diabetes at Risk of Uncontrolled Outcomes during a Diabetes Management Program: Conceptualization and Development Study Using Machine Learning
Source: JMIR Form Res. 2024 Apr 26;8:e54373. doi: 10.2196/54373 (PMC11087850; doi:10.2196/54373)
Supplement: Multimedia Appendix 1 [file formative_v8i1e54373_app1.pdf]

MULTIMEDIA APPENDIX 1

Multimedia Appendix 1. Number of participant cases and controls for each data subset

Journey month	Dataset Subset	Observable		Unobservable	
		Cases, %	Controls, %	Cases, %	Controls, %
0	train	22.3%	77.7%	26.3%	73.7%
0	test	22.3%	77.7%	26.0%	74.0%
1	train	21.2%	78.8%	26.6%	73.4%
1	test	20.9%	79.1%	27.0%	73.0%
2	train	21.8%	78.2%	29.0%	71.0%
2	test	21.7%	78.3%	29.4%	70.6%
3	train	22.1%	77.9%	30.7%	69.3%
3	test	21.9%	78.1%	31.7%	68.3%
4	train	22.2%	77.8%	31.6%	68.4%
4	test	22.0%	78.0%	32.9%	67.1%
5	train	22.1%	77.9%	32.1%	67.9%
5	test	21.9%	78.1%	33.2%	66.8%
6	train	21.9%	78.1%	32.4%	67.6%
6	test	21.7%	78.3%	33.5%	66.5%
7	train	21.8%	78.2%	32.9%	67.1%
7	test	21.7%	78.3%	33.3%	66.7%
8	train	21.8%	78.2%	33.5%	66.5%
8	test	21.7%	78.3%	34.1%	65.9%
9	train	22.1%	77.9%	33.6%	66.4%
9	test	21.9%	78.1%	34.6%	65.4%
10	train	22.4%	77.6%	33.3%	66.7%
10	test	22.3%	77.7%	34.4%	65.6%
11	train	22.9%	77.1%	33.2%	66.8%
11	test	22.9%	77.1%	32.9%	67.1%
