# Supplementary material for: Proactive Identification of Patients with Diabetes at Risk of Uncontrolled Outcomes during a Diabetes Management Program: Conceptualization and Development Study Using Machine Learning
Source: JMIR Form Res. 2024 Apr 26;8:e54373. doi: 10.2196/54373 (PMC11087850; doi:10.2196/54373)
Supplement: Multimedia Appendix 3 [file formative_v8i1e54373_app3.pdf]

MULTIMEDIA APPENDIX 3

Multimedia Appendix 3. Performance metrics for each data subset for unobservable members

Month in Program Journey	Subset	Recall	Specificity	Precision	AUC	F_1 Score	Accuracy
0	train	0.641	0.687	0.422	0.73	0.509	0.675
0	test	0.616	0.678	0.402	0.703	0.486	0.662
1	train	0.811	0.725	0.516	0.85	0.631	0.748
1	test	0.789	0.719	0.51	0.835	0.619	0.738
2	train	0.783	0.7	0.516	0.823	0.622	0.724
2	test	0.745	0.692	0.502	0.795	0.6	0.707
3	train	0.801	0.74	0.577	0.848	0.67	0.759
3	test	0.684	0.692	0.507	0.754	0.582	0.689
4	train	0.783	0.717	0.561	0.835	0.654	0.738
4	test	0.729	0.707	0.549	0.791	0.626	0.714
5	train	0.784	0.72	0.57	0.831	0.66	0.741
5	test	0.716	0.695	0.539	0.785	0.615	0.702
6	train	0.784	0.739	0.59	0.842	0.673	0.753
6	test	0.739	0.717	0.568	0.802	0.643	0.725
7	train	0.852	0.784	0.659	0.898	0.743	0.807
7	test	0.763	0.741	0.595	0.833	0.669	0.749
8	train	0.806	0.758	0.626	0.863	0.705	0.774
8	test	0.741	0.726	0.583	0.821	0.653	0.731
9	train	0.847	0.794	0.676	0.902	0.752	0.812
9	test	0.807	0.763	0.643	0.869	0.716	0.778
10	train	0.866	0.793	0.677	0.907	0.76	0.817
10	test	0.806	0.761	0.638	0.863	0.713	0.776
11	train	0.876	0.805	0.691	0.914	0.772	0.829
11	test	0.822	0.748	0.615	0.861	0.704	0.772
